# Association between Dairy Intake and Gastric Cancer: A Meta-Analysis of Observational Studies

**DOI:** 10.1371/journal.pone.0101728

**Published:** 2014-07-09

**Authors:** Shu-bo Tian, Jian-chun Yu, Wei-ming Kang, Zhi-qiang Ma, Xin Ye, Zhan-jiang Cao

**Affiliations:** Department of General Surgery, Peking Union Medical College Hospital, Peking Union Medical College, Chinese Academy of Medical Sciences, Beijing, China; NIH - National Institute of Environmental Health Sciences, United States of America

## Abstract

**Purpose:**

Observational studies have given inconsistent findings on the relationship between intake of dairy products and gastric cancer. We therefore conducted a systematic review with a meta-analysis of observational studies to summarize available evidence on this point.

**Methods:**

We searched the electronic literature databases of PubMed (Medline), EMBASE and the Chinese Biomedical Literature Database up until August 30, 2013. All studies were limited to the English language. Random-effects models were used to pool study results between dairy products consumption and the risk of gastric cancer. We also performed subgroup, publication bias and sensitivity analysis.

**Results:**

Eight prospective studies and 18 case-control studies were included in our analysis, with a total number of 7272 gastric cancer cases and 223,355 controls. Pooled relative risks of all studies showed no significant association between dairy intake and gastric cancer (odds ratio [OR]: 1.09, 95% confidence interval [CI]: 0.96–1.25). When study design was separately analyzed, population-based case-control studies showed a positive association between dairy intake and gastric cancer risk (OR: 1.36; 95% CI: 1.07–1.74), whereas no associations were shown by hospital-based case-control studies (OR: 0.86, 95% CI: 0.72–1.02) or cohort studies (OR = 1.01, 95% CI = 0.91–1.13).

**Conclusions:**

The meta-analysis shows that no clear association apparently exists between consumption of dairy products and gastric cancer risk. Further well-designed cohort and intervention studies should be conducted to verify this lack of association.

## Introduction

Gastric cancer is a major global health problem. Global data in 2008 showed that the standardized incidence of gastric cancer was located in 6th of all tumors [1]. Overall incidence and mortality of gastric cancer have decreased in recent decades [2], which is mainly attributed to improved treatment for *H. pylori*, the primary risk factor for gastric cancer. However, gastric cancer is rarely diagnosed early, and is usually surgically unresectable when detected. Dietary intake may affect its development; some studies have found that high intake of dietary fiber, alliums and crucifers, and lower dietary salt intake could reduce the risk of gastric cancer [3].Milk and dairy products are important components of diets in some but not all parts of the world. They contain a number of vitamins and minerals, including retinol, riboflavin, and calcium. The relationship between dairy intake and cancer has been widely studied. Whereas milk apparently protects against colorectal cancer, a meta-analysis associated milk and dairy products, but not cheese, with reduced colorectal cancer risk [4]. Some system reviews associated high intake of dairy products with increased risk of prostate cancer [5]–[6], another meta-analysis of prospective cohort studies associated higher total dairy product intake with reduced breast cancer risk [7]. Several prospective studies have also related higher milk consumption with increased risk of ovarian cancer [8]–[9]. However, conclusions vary on the association between dairy intake and risk of gastric cancer. Although several epidemiologic studies investigated this relationship, both direct and inverse associations have been reported between dairy product intake and gastric cancer risk. This meta-analysis evaluated the relationship between dairy consumption and risk of gastric cancer. We also examined dairy intake in relation to gastric cancer risk according to study designs, geographic area, gastric cancer subtypes and gender. To our knowledge, this is the first meta-analysis to investigate associations between dairy or milk intake and gastric cancer with diverse studies conducted in different countries.

## Methods

### Search strategy

We conducted a comprehensive search of PubMed (Medline), EMBASE and the Chinese Biomedical Literature Database for both case-control and cohort studies that evaluated effects of dairy products consumption on the risk of gastric cancer. The studies were published in English, and dated up to August 2013. References from the selected studies were also searched. The following search strategy was used: (gastric cancer OR stomach cancer) combined with dairy (milk, cheese, creams). Studies were included in the meta-analysis if they presented data on the association between dairy consumption and gastric cancer.

### Study selection criteria

Two investigators (S.-B.T. and Z.-J.C) evaluated the eligibility of all retrieved studies and extracted the relevant data independently. Disagreement was resolved by discussion. Studies included in the meta-analysis met the following criteria: (1) case-control or cohort design; (2) diagnoses were histologically confirmed by pathologists; (3) evaluated association between dairy products (milk, cheese, yogurt) and gastric cancer risk; and (4) presented OR, relative risk (RR), or hazard ratio (HR) estimates with its 95%CI. If a study provided several ORs or RRs, we extracted the ORs or RRs that were most fully adjusted for potential confounders.

### Data extraction

Data extracted from studies included: first author's name, publication year, study design, dairy product type, and OR or RR with corresponding 95% CI for each category. Studies were also assessed by the Newcastle–Ottawa Scale [10], which evaluates observational studies from three aspects: study group selection, group comparability, and determination of exposure or outcome of interest for case-control or prospective studies, respectively. The full score was 10 and a high quality study was defined as one with quality ≥7.

### Statistical methods

Our main analysis focused on associations between consumption of total dairy products or milk and gastric cancer. Because the absolute risk of gastric cancer is low, so the HR and RR were taken as approximations of OR; we therefore report all results as the OR for simplicity. If studies reported separate results for cardia and non-cardia, intestinal and diffuse cancer, or men and women patients, we combined the two results using a fixed-effects model to obtain an overall combined estimate before pooling with other studies; ORs were similarly pooled for studies that described results of several dairy products. The studies included in our meta-analysis used different units to report dairy products intake (eg., grams, glasses and times). So, the cutoffs for high and low exposure categories varied across studies included in our meta-analysis. The definitions of “high vs. low categories of total dairy consumption” of all studies were presented in the Table S1.

We used the random-effect model to calculate combined risk estimate. We assessed heterogeneity with *I* ^2^ and Cochran *Q* statistics[11], which quantitatively measure inconsistency across studies. For Q statistics, a *P*<0.1 was considered to indicate substantial heterogeneity. *I* ^2^ represents the proportion of total variation contributed between studies. *I* ^2^>50% is suggestive of considerable heterogeneity. Subgroup analyses by study characteristics were conducted to investigate potential sources of heterogeneity. Subgroup analyses were stratified by study design (cohort or case-control), region (Asia, Europe, North America and South America), cancer subsite (cardia or non-cardia), Lauren's classification (diffuse or intestinal) and dairy type (milk, cheese or yogurt).

In a sensitivity analysis of dairy intake and gastric cancer, we sequentially excluded each study one by one and re-analyzed the data. Potential publication bias was assessed by both Begg rank correlation and Egger linear regression test [12], in which the standard error (SE) of log(OR) of each study was plotted against log(OR), with the following formula: SelogOR  =  (logUCI – logLCI)/3.92; where UCI and LCI represents the upper and lower 95% CI limits, respectively, for OR. *P*<0.05 for Egger's or Begg's tests was considered to indicate significant publication bias. In addition, meta-regression analyses were conducted to investigate potential sources of heterogeneity. We used the Stata statistical program, version 12.0 (StataCorp) for analyses. *P*-values were two-sided, and considered significant at *P*<0.05.

## Results

### Literature search

**Figure 1:**
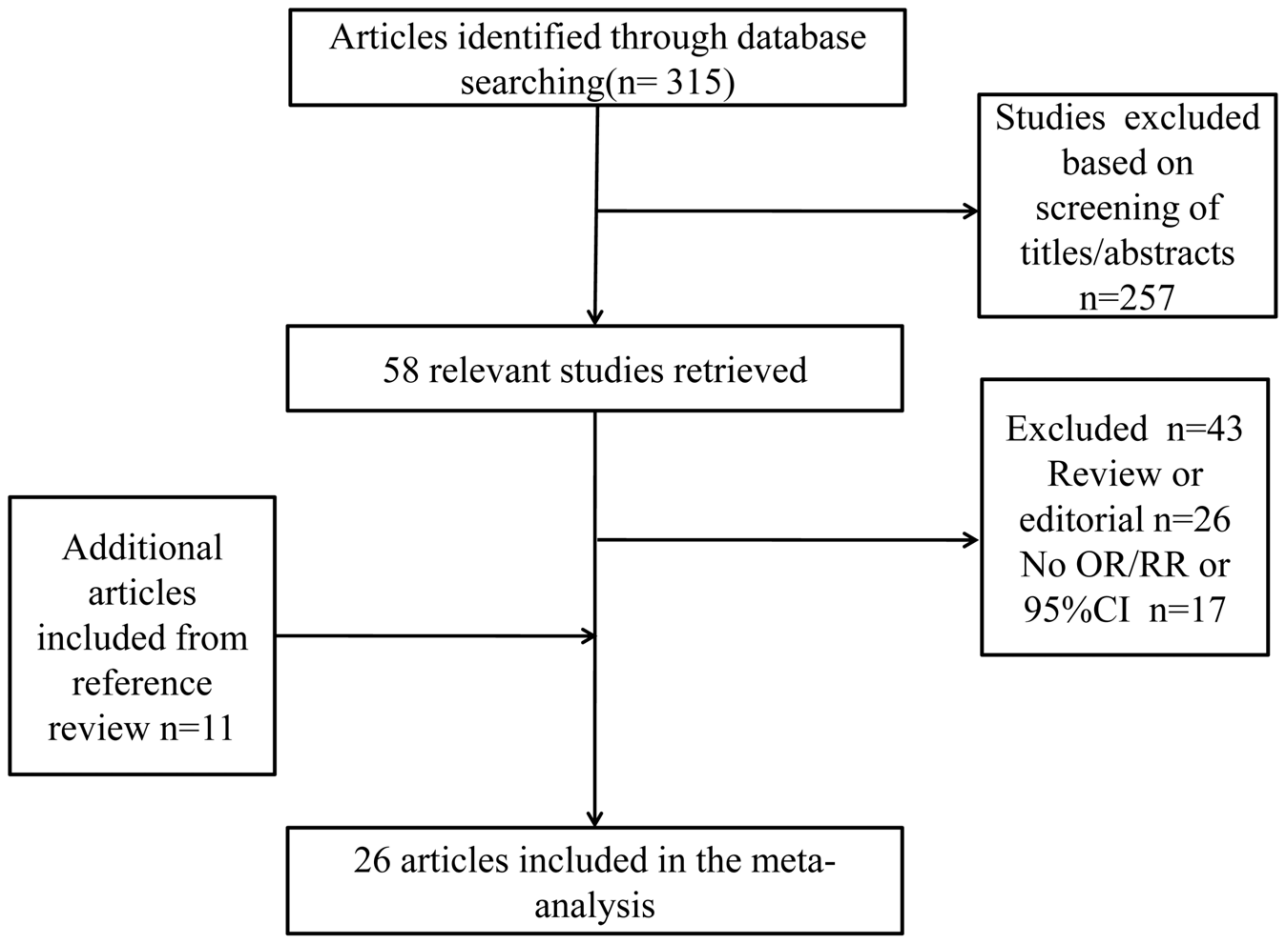
Flow chart of the included studies.

We identified 315 potentially relevant articles from our search of the three databases. Of these, 257 articles were excluded after screening titles or abstracts, leaving 58 articles for full-text review. Hand-searching reference lists of reviews and retrieved articles identifying the relevant studies. The flow chart of the literature search was shown in Figure 1. After exclusion, only 26 studies on dairy consumption and gastric cancer incidence were included in the meta-analysis [13]–[38]. Characteristics of included studies are summarized in Table S1. The quality scores of each study are summarized in Table1 and Table2. The quality scores ranged from 5 to 9.

**Table 1:** Methodologic quality of case-control studies included in the Meta-analysis.

Study(The first author and published year)	Adequate definition of cases	Representativeness of cases	Selection of controls	Definition of controls	Control for important factors a	Exposure assessment^ b^	Same method of ascertainment for all subjects	Nonresponse rate	Total quality scores
Boeing H et al, 1991	☆	☆	—	☆	☆☆	☆	☆	—	7
Boeing H et al, 1991	☆	☆	—	☆	☆☆	—	☆	—	6
Hansson LE,1993	☆	☆	☆	—	☆☆	☆☆	☆	☆	9
Cornee J,1995	☆	☆	—	☆	☆☆	—	☆	—	6
Harrison LE, 1997	☆	☆	—	☆	☆☆	—	☆	—	6
Ward MH, 1999	☆	☆	☆	—	☆☆	—	☆	☆	7
Muñoz N et al, 2001	☆	—	—	☆	☆☆	—	☆	—	5
Kim HJ et al, 2002	—	☆	☆	—	☆☆	☆	☆	☆	7
Chen H et al, 2002	☆	☆	☆	—	☆☆	☆	☆	☆	8
Ito LS, 2002	☆	☆	—	☆	☆☆	—	☆	—	6
De Stefani E, 2004	☆	☆	—	☆	☆☆	☆	☆	—	7
Lissowska J, 2004	☆	☆	☆	—	☆☆	—	☆	☆	7
Fei SJ, 2006	☆	☆	—	☆	—	☆	☆	—	5
Navarro Silvera SA, 2008	☆	☆	☆	—	☆☆	☆☆	☆	☆	9
Pourfarzi F, 2009	☆	☆	☆	—	☆☆	☆	☆	☆	8
Lazarevic K, 2010	☆	☆	—	☆	☆☆	—	☆	—	6
Gao Y, 2011	☆	☆	☆	—	☆☆	—	☆	—	6
Pakseresht M, 2011	☆	☆	☆	—	☆☆	—	☆	☆	7

Note: A study can be given a maximum of one star for each item with the Selection and Outcome categories. A maximum of two stars can be awarded for Comparability. (Column 2–5, Selection; Column 6, Comparability; Column 7–9, Outcome).

a A maximum of 2 stars awarded for this item. Studies that controlled for age received 1 star, and studies that controlled for other factors received an additional star. ^b^ A maximum of 2 stars awarded for this item. Studies that ascertained the exposure with a secure record received 1 star, and studies with structured interview where blind to case/control status received an additional star.

**Table 2:** Methodologic quality of cohort studies included in the Meta-analysis.

First author (year of publication)	Representativeness of the exposed cohort	Selection of the nonexposed cohort	Ascertainment of expose	Outcome of interest not present at start of study	Control for important factors a	Assessment of outcome	Follow-up period long enough for outcomes to occur b	Adequacy of follow-up evaluation of cohorts c	Total quality scores
Nomura A,1990	☆	☆	☆	☆	☆	☆	☆	☆	8
Kneller RW, 1991	☆	☆	☆	☆	☆☆	☆	☆	☆	9
Galanis DJ,1998	☆	☆	☆	☆	☆☆	☆	☆	☆	9
Ngoan L, 2002	☆	☆	☆	☆	☆☆	☆	☆	☆	9
Khan MM, 2004	☆	☆	☆	☆	☆☆	☆	☆	☆	9
Tokui N, 2005	☆	☆	☆	☆	☆	☆	☆	—	7
van der Pols JC, 2007	☆	☆	☆	☆	☆	☆	☆	☆	8
Ko kp, 2013	☆	☆	☆	☆	☆☆	☆	☆	—	8

Note: A study can be given a maximum of one star for each item with the Selection and Outcome categories. A maximum of two stars can be awarded for Comparability. (Column 2–5, Selection; Column 6, Comparability; Column 7–9, Outcome).

a A maximum of 2 stars could be awarded for this item. Studies that controlled for age received 1 star, and studies that controlled for smoking received an additional star.

b A cohort study with a follow-up time longer than 5 years was assigned 1 star.

c A cohort study with a follow-up rate >70% was assigned 1 star.

### Study characteristics and quality assessment

Eight of these studies were cohort studies and 18 were case-control studies. Geographically, 10 were carried out in Asia, seven in Europe, seven in North America, and two in South America. Most studies were adjusted for a wide range of confounders, including age, sex, body mass index (BMI), smoking and alcohol drinking.

### High vs. low categories of total dairy consumption and gastric cancer risk

**Figure 2:**
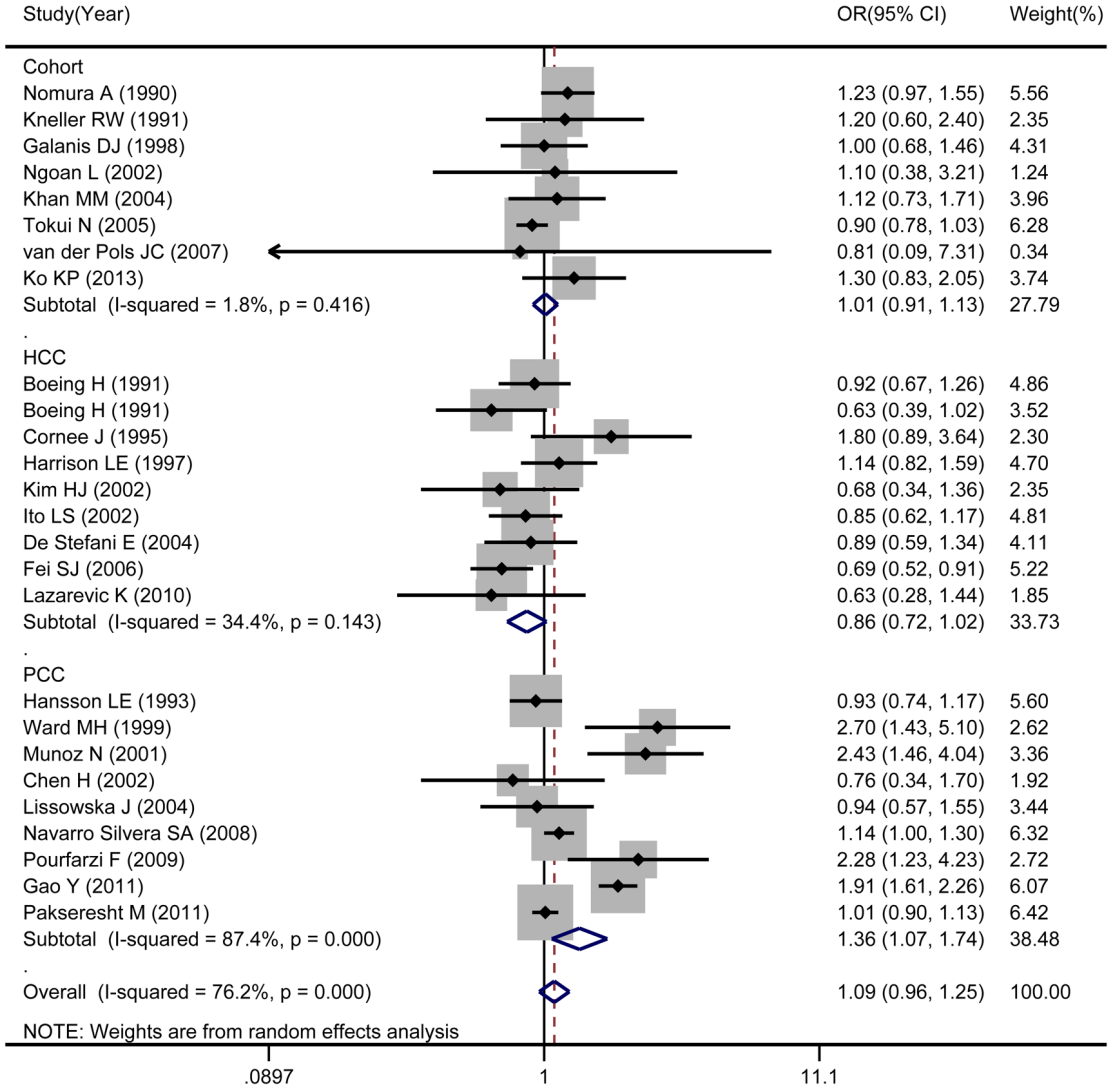
Consumption of total dairy foods in association with gastric cancer.

Combined results based on all studies indicated that total dairy consumption was not associated with gastric cancer risk (OR: 1.09; 95% CI: 0.96–1.25; Figure 2). However, we detected significant heterogeneity (*I* ^2^: 76.2%; Q: 105.22; *P*<0.001) among these studies.

In subgroup analysis by study design, the combined risk estimate was 1.01(95% CI: 0.91–1.13) for cohort studies, 0.86 for hospital-based case-control studies (95% CI: 0.72–1.02). However, when population-based case-control studies were combined, pooled OR for total dairy intake showed a positive, significant association with rate of gastric cancer. High versus low dairy product intake correlated with a 36% increase in risk of gastric cancer (OR: 1.36, 95% CI: 1.07–1.74) in this subgroup.

Of the 26 studies we analyzed, seven provided separate data on women and men. Two prospective studies examined the relationship between stomach cancer and dairy intake in men only, and one retrospective study investigated the association in women only. Results of stratified analysis by gender showed that dairy product or milk consumption was not significantly associated with gastric cancer in men (OR: 1.04; 95% CI: 0.96–1.13) or women (OR: 1.02; 95% CI: 0.95–1.09).

To explore potential heterogeneity, subgroup analyses were also conducted by geographic area, Lauren's classification and location of gastric cancer, but these analyses did not show significant effects of dairy products or milk intake on gastric cancer incidence (Table 3). By geographic area, analysis of North Americans gave an OR of 1.18 (95% CI: 1.01–1.38), whereas studies of Europeans showed a tendency toward an inverse relationship between dairy consumption and gastric cancer risk (OR: 0.91; 95% CI: 0.76–1.08). OR was 1.09 (95% CI: 0.86–1.38) for the 10 Asian studies, and 1.45 (95% CI: 0.54–3.89) for the two studies from South America. The summary OR of two cohort studies conducted in Hawaii (population of Japanese ancestry) was 1.11 (95% CI: 0.85–1.44).

**Table 3:** Stratified Analysis of dairy products or milk Intake and gastric cancer Risk.

Group	NO. of studies	OR(95%CI)	Q statistic	P value	I^2^ value(%)
Total Dairy products	26	1.09(0.96–1.25)	105.22	<0.001	76.2
Design					
Cohort	8	1.01(0.91–1.13)	7.13	0.416	1.8
Case-control	18	1.10(0.92–1.30)	95.42	<0.001	82.2
Hospital based	9	0.86(0.72–1.02)	12.19	0.143	34.4
Population based	9	1.36(1.07–1.74)	63.26	<0.001	87.4
Geographic area					
Asia	10	1.09(0.86–1.38)	72.34	<0.001	87.6
Europe	7	0.91(0.76–1.08)	6.63	0.356	9.6
North America	7	1.18(1.01–1.38)	8.73	0.189	31.3
South America	2	1.45(0.54–3.89)	9.14	0.003	89.1
Lauren's classification					
Diffuse	4	1.04(0.87–1.24)	2.58	0.461	0
Intestinal	4	1.13(0.79–1.62)	9.15	0.027	67.2
Location					
Cardia	3	1.32(0.84–2.08)	25.74	<0.001	92.2
Noncardia	3	1.21(0.95–1.54)	10.98	0.004	81.8
Gender					
Men	9	1.04(0.96–1.13)	13.19	0.587	0
Women	8	1.02(0.95–1.09)	10.59	0.751	0
Adjustment for confounders					
Alcohol Yes	7	1.18(0.96–1.25)	10.65	0.1	43.6
No	19	1.18(0.98–1.42)	92.75	<0.001	80.6
Smoking Yes	15	1.10(0.96–1.25)	27.34	0.017	48.8
No	11	1.07(0.83–1.39)	77.73	<0.001	87.1
BMI Yes	5	1.13(1.01–1.26)	1.71	0.798	0
No	21	1.09(0.93–1.29)	102.92	<0.001	80.6
Total energy intake Yes	10	1.22(1.02–1.46)	22.83	0.007	60.6
No	16	1.01(0.83–1.23)	82.11	<0.001	81.7
H. pylori infection Yes	2	1.43(0.65–3.15)	6.46	0.011	84.5
No	24	1.08(0.93–1.25)	98	<0.001	76.5
Publication year					
Before 2000	9	1.10(0.90–1.34)	18.83	0.016	57.4
After 2000	17	1.08(0.91–1.29)	86.30	<0.001	81.5
Quality score					
<7 stars	8	1.11(0.75–1.65)	65.81	<0.001	89.4
≥7 stars	18	1.05(0.95–1.16)	28.70	0.037	40.8
Fermented(Yes vs. No)					
Non-Fermented	13	1.10(0.92–1.32)	30.68	0.01	51.1
Fermented	8	0.94(0.81–1.08)	15.42	0.42	2.7
Milk					
Asia	5	0.90(0.75–1.08)	7.64	0.501	0
Europe	5	1.57(1.01–2.44)	8.71	0.126	51.8
North America	3	1.68(0.90–3.14)	4.15	0.069	54.1
Hawaii	2	1.11(0.85–1.44)	0.45	0.106	47.6
Cheese					
Asia	3	1.05(0.72–1.54)	0.35	0.840	0
Europe	4	0.75(0.52–1.08)	9.19	0.027	67.4

**Figure 3:**
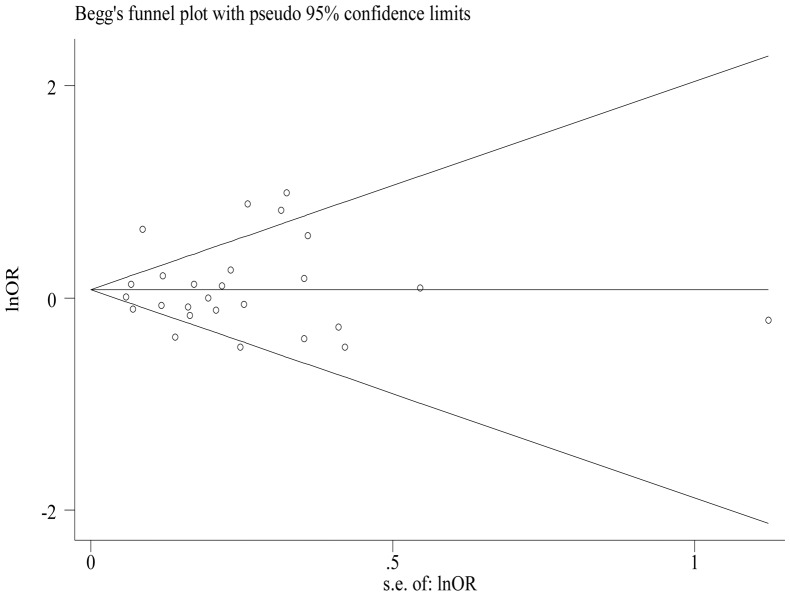
Funnel plot of studies evaluating the association between gastric cancer and total dairy consumption.

Non-Fermented: milk; Fermented: cheese, yogurt, sour milk;

No publication bias was detected by Begg's rank correlation test (*P* = 0.343; Figure 3.) or by Egger's regression test (*P* = 0.889), nor did a funnel plot suggest asymmetry distribution.

### Milk and gastric cancer risk

**Figure 4:**
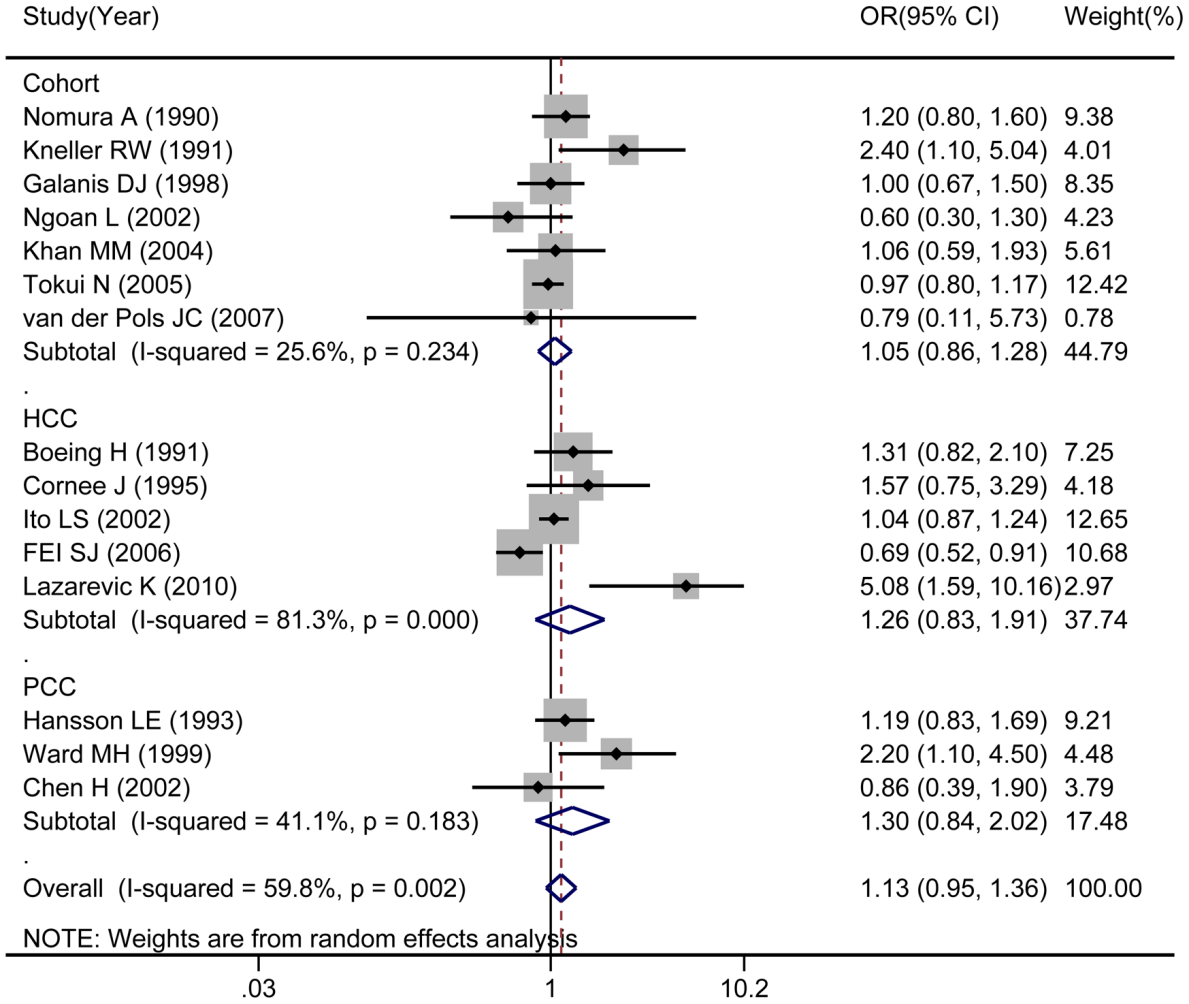
Consumption of milk in association with gastric cancer.

Milk consumption was examined in seven cohort studies, five hospital-based case-control studies and three population-based studies. Combined results based showed that milk consumption was not associated with gastric cancer risk (OR: 1.13; 95% CI: 0.95–1.36; Figure 4). Subgroup analyses by study design, cohort studies (OR: 1.05, 95%: 0.86–1.28), hospital-based case-control studies (OR: 1.26; 95% CI: 0.83–1.91), and population-based case-control studies (OR: 1.30; 95% CI: 0.84–2.02) yielded similar results. None of these group studies showed significant association between milk consumption and gastric cancer risk. Subgroup analysis on geographic areas, studies in Europe associated higher milk consumption with increase risk of gastric cancer (OR: 1.57; 95% CI: 1.01–2.44). All studies were significantly heterogeneous (*I* ^2^ = 59.8%, *P* = 0.002). No publication bias was detected in either analysis by the tests of Begg (P = 0.166) or Egger (P = 0.112).

### Cheese and risk of gastric cancer

**Figure 5:**
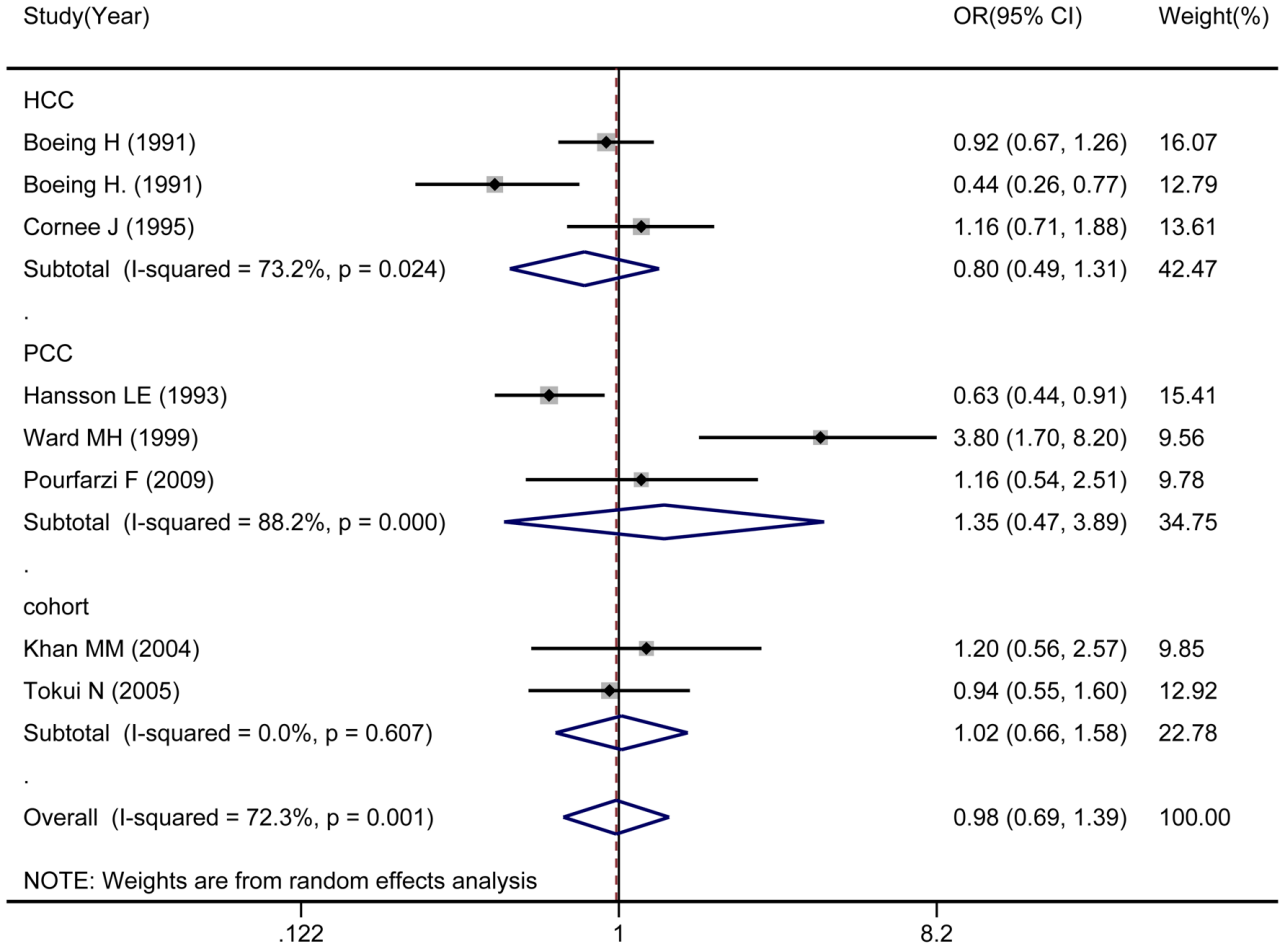
Consumption cheese in association with gastric cancer.

Eight studies examined the association between cheese intake and risk of gastric cancer. High cheese intake versus low intake was not significantly associated with gastric cancer risk (OR: 0.98; 95% CI: 0.69–1.39; Figure 5), nor was any significant association found in subgroup analysis by study design. OR was 0.80 (95% CI: 0.49–1.31) for hospital-based case-control studies, 1.35 (95% CI: 0.47–3.89) for population-based case-control studies, and 1.02 (95% CI: 0.66–1.58) for cohort studies. In the subgroup analysis by geographic area, no association was observed in studies conducted in Asia and Europe. There was statistically significant heterogeneity for studies included in Europe between cheese intake and gastric cancer risk(*I* ^2^ = 67.4%, *P* = 0.027). Publication bias was detected in neither analysis (Begg's test, P = 0.386; Egger's test, P = 0.229).

We also carried out a meta-analysis of the relationships between the consumption of fermented (cheese, yogurt) and non-fermented (milk) dairy products and gastric cancer risk. No significant association was found between fermented and non-fermented dairy consumption and gastric cancer (Table 3).

### Sensitivity analysis and meta-regression analysis

**Figure 6:**
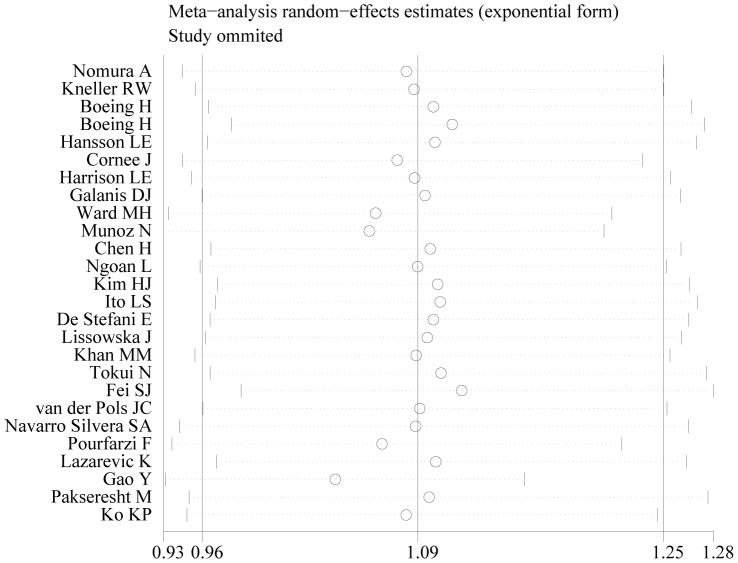
Sensitivity analysis of total dairy intake and gastric cancer risk.

In sensitivity analysis of total dairy intake and gastric cancer risk, the 26 study-specific ORs ranged from a low of 1.04 (95% CI: 0.93–1.16) to a high of 1.12 (95% CI: 0.98–1.28) via omission of the studies by Gao et al [36] and Fei et al [31] (Figure 6). As shown in Table 4, meta-regression analysis confirmed that study design might be a main source of heterogeneity. PCC was found to be the possible influence factors. Neither publish year or geographic area was responsible for the between-study heterogeneity.

**Table 4:** Meta-regression Analysis.

Covariant	Coefficient	Standard error	P value	95%CI
Publication Year	0.007	0.011	0.527	−0.015 to 0.029
Geographic area (Ref = South America)				
Asian	−0.261	0.296	0.389	−0.878 to 0.356
European	−0.445	0.316	0.173	−1.102 to 0.212
North America	−0.118	0.325	0.720	−0.795 to 0.558
Hawaii	−0.231	0.366	0.535	−0.992 to 0.530
Study design (Ref = HCC)				
Cohort	0.234	0.165	0.170	−0.108 to 0.575
PCC	0.443	0.152	0.008	−0.130 to 0.757

PCC, Population-based Case-Control; HCC, Hospital-based Case-Control; Ref, Reference.

## Discussion

This meta-analysis summarizes results of observational studies, including 26 studies published during the last two decades, with a total of 7272 cases and 223,355 controls, and found that high dairy products or milk consumption was not associated with gastric cancer risk, and total dairy food intake did not apparently affect prevention of gastric cancer. Specific analyses for cheese yielded similar results.

The findings of the meta-analysis of population-based case-control studies indicated that increased consumption of total dairy food is positively associated with the risk of gastric cancer. Although this result is inconsistent with the cohort studies and hospital-based case-control studies, such conflicting results are often seen in cancer epidemiologic studies, as case-control studies use subjects' histories before their cancer diagnoses, which may be unreliable. In addition, all case-control studies show selection, information and confounding biases as a result of their retrospective nature. Subjects with gastric cancer may have consumed more milk and dairy products to relieve gastric pain before their diagnoses [35], so higher consumption may reflect recent changes in diet, and thus be a consequence of, rather than a reason for, gastric cancer. However, the meta-analysis of hospital-based case-control studies showed dairy food intake was inversely associated with gastric cancer incidence, although not significantly so. Cohort studies are thought to be more rigorous than case-control studies for identification and evaluation of risk factors associated with a disease. They can reduce selection bias and recall bias and better explain etiological connections. However, cohort studies in this meta-analysis showed no relationship between dairy products or milk consumption and gastric cancer.

We conducted subgroup analyses to determine the impact of differences in study design, geographic area and dairy food types on our study. Results from stratified analyses by gender, Lauren's classification and anatomic subsite were similar to those from our analysis of the 26 observational studies. However, when stratifying by geographic area, dairy intake was significantly associated with gastric cancer in North America. This significant risk was largely attributable to research conducted by Ward et al. in Mexico [21], which associated an increased risk with frequent consumption of dairy products (OR: 2.7; 95% CI: 1.4–5.0); this result of subgroup analysis for North America should therefore be considered with some caution. After adjustment for BMI and total energy intake, dairy consumption showed a slight promotion effect on developing stomach cancer. High intake of total energy correlates with increased BMI, which is the most commonly used measure of excess body weight and obesity. A meta-analysis of prospective cohort studies demonstrated high energy intake may increase the risk of gastric cancer [39]. Another meta-analysis of BMI and gastric cardia cancer showed a positive association [40]. The mechanism underlying this association is thought to be increased production of insulin and insulin-like growth factor-1(IGF-1) from accumulated adipose tissue. The IGF-1 signaling pathway has been implicated in gastric cancer development. Binding of IGF-1 to receptor IGF1R activates the pathway and enhances tumor development by stimulating cell proliferation and inhibiting apoptosis [41]. However, the OR in this study after adjusting for BMI is 1.13 (95% CI: 1.01–1.26); the OR is 1.22 (95% CI: 1.02–1.46) after adjusting for total energy. BMI and total energy intake may confound the dairy effects. The results call for more clinical trials.

Although some observational studies associate adequate intake of dairy products or milk with low incidence of gastric cancer, other studies have documented a positive relation. A meta-analysis published in 2008 showed that dairy product consumption might decrease risk of gastric cancer [42], but it only included eight studies, all of them case-control studies conducted in China. In comparison, our analysis yielded a nonsignificant conclusion. After all, there are discrepancies of dietary habit between the Western countries and China. People in high-income countries always consume more cows' milk and its products. In China, milk and dairy accounted for only 0.3% of total energy in Chinese people in the 30 years from 1964 to 1994, but about 10% of that in Western countries [43]. Consumption of milk and dairy products increases linearly in China, but is still less than in Western countries. However, China has a high incidence of gastric cancer. Although dairy or milk intake can apparently decrease the risk of gastric cancer to some extent, Chinese and Western people differ greatly in their dietary habits, so we cannot conclude that dairy products or milk consumption could prevent gastric cancer. Among Asians, dietary habits between Chinese and Japanese people are similar. Two studies were conducted by Nomura et al [13] and Galanis et al [20] among Japanese residents of Hawaii, a high-risk population for gastric cancer in the United States, whose dietary habits might change after migrating from Japan to Hawaii. The two studies showed no association between the incidence of gastric cancer and dairy products or milk consumption.

Research that suggests that milk or dairy products can prevent incidence of gastric cancer often uses a theory that whole milk and dairy products have high proportions of energy from fat and protein, and contain some vitamins and minerals. Some fatty acids present in the milk have antitumor effects, the best-known being conjugated linoleic acid (CLA). CLA has been shown to inhibit in vitro proliferation of human gastric cancer cells, and prevent development of forestomach cancer in mice [44]. Other components in milk fat, such as sphingomyelin [45], butyric acid [46] and ether lipids [47], also showed potential antitumor activity against human cancer cells in vitro experiments.

Epidemiological studies have revealed a higher risk of gastric cancer in people who consume milk from livestock that have fed on bracken fern [48]. The main chemical constituent, ptaquiloside, which was extracted from bracken fern, has been shown to induce gastric cancers in experimental animals. Some studies provided direct evidence that this carcinogen could induce genetic instabilities and DNA damage response in gastric cancer cells in vitro and in a mouse model [49]-[50]. Other studies also showed that bracken fern have immunosuppressive effects and modulate many functions, thus possibly contributing to an increased risk of gastric cancer formation [51]. On the other hand, milk also contains several growth factors and hormones, although most of these components are digested in the stomach. However, milk consumption increases serum IGF-1, which has been associated with increased gastric cancer. Patients with gastric cancer may show serum IGF-1 levels significantly increased over normal limits [52].

Strengths of our study were the large number of participants and events, which both made abundant data available and facilitated analyses of many subgroups. However, our limitations include, first, unmeasured or uncontrolled confounding factors inherited from the original studies. All risk estimates were derived from multivariable models, but individual studies did not adjust for potential risk factors in a consistent way. Second, we note some misclassification bias. Inevitably, dietary assessments suffer from measurement error, and food frequency questionnaires and other methods used to report dairy food or milk intake differed among these studies. Third, the studies showed substantial heterogeneity, which likely reflected variation in sample size, exposure definitions, assessment of outcomes, categorization of dairy intake, and population characteristics. Therefore, this result should be considered with some caution because of exposure misclassification and different ranges of dairy products intake. Fourth, because of different methods used to assess and report dairy intake across studies, we failed to evaluate a dose-response relation between dairy food intake and gastric cancer. Fifth, as studies included in our meta-analysis were of cohort or case-control design, the possibility of recall and selected biases cannot be excluded. Cohort studies are less susceptible to bias, but showed similar results to case-control studies, indicating that recall and selection biases did not greatly affect the findings. Sixth, only two studies included in our meta-analysis had information on *H. pylori* infection, which is a key risk factor for gastric cancer. Summary ORs of these two studies showed a nonsignificant 43% increased risk of gastric cancer after adjustment for *H. pylori* infection. Fermented dairy products such as cheese and yogurt contain lactic acid bacteria(LAB) which have been shown to be an effective chemopreventive food ingredient against gastric cancer. The mechanism is that LAB can suppress growth of H. pylori by producing inhibitory substances such as lactic acid and bacteriocin, or compete for gastric epithelial cell surface and mucin binding sites [53]. In vitro experiments also showed that lactic acid bacteria extracts could inhibit the bacterial adhesion and invasion, gastric inflammation induced by *H. pylori* [54]. However, in our meta-analysis, no significant association was found between fermented dairy or non-fermented dairy consumption and gastric cancer. Further investigations with appropriate adjustment for this confounding factor are warranted to evaluate the role of dairy or milk in gastric cancer etiology.

In conclusion, our analysis indicates that dairy intake is not significantly associated with gastric cancer. Although we find that dairy products are unlikely to be strongly protective against gastric cancer, they are not adverse factors. Because of the complexity of both milk components and gastric cancer, evaluating the effect of milk on this disease is difficult. Some materials, such as IGF-1, can promote gastric cancer. Other materials, such as calcium, vitamin D and CLA, protect against gastric cancer. Thus the overall effect of dairy products in gastric cancer is not obvious in humans. Moreover, although the promotion and protection effects of these substances have been verified at molecular and cellular levels, they lack strong support in epidemiological studies. Of course, we cannot ignore the huge nutritive value of dairy products, which contributes tremendously to human health. In addition, human experimental studies on associations between dairy intake and gastric cancer are unlikely, so observational studies are the best available source of evidence on risk. Future studies need to account more accurately for long-term dairy product intake. More in-depth studies, with strong dietary assessment tools and appropriate adjustment for confounding factors, should be conducted to evaluate the role of dairy products and milk in gastric cancer etiology.

## Supporting Information

Table S1 Characteristics of studies of dairy products or milk intake and gastric cancer risk.
(DOC)

Checklist S1 PRISMA 2009 checklist.
(DOC)
