# Supplementary material for: Association between Dairy Intake and Gastric Cancer: A Meta-Analysis of Observational Studies
Source: PLoS One. 2014 Jul 9;9(7):e101728. doi: 10.1371/journal.pone.0101728 (PMC4090187; doi:10.1371/journal.pone.0101728)
Supplement: Table S1 — Characteristics of studies of dairy products or milk intake and gastric cancer risk. (DOC) [file pone.0101728.s001.doc]

**Table S1****. Characteristics of studies of dairy products or milk intake and gastric cancer risk**

| **Study(first author and year of publication)** | **Study design** | **gender** | **Study location** | **No. of Cases/**  **controls** | **Dietary assessment/**  **method of assessment of dairy source** | **High vs. low categories of dairy intake** | **Dairy source** | **Adjusted OR(95%CI) highest vs. lowest intake categories** | **Adjustment** |
| --- | --- | --- | --- | --- | --- | --- | --- | --- | --- |
| Nomura A et al,1990^[9]^ | Cohort | Man | United States (Hawaii) | 150/7990 | FFQ/First Health and Nutrition Survey | Tertile (≥5/wk vs.≤1/wk) | Milk  Ice cream  Butter Cheese | 1.20(0.80-1.60)  1.10(0.70-1.80)  1.40(0.90-2.20) | Age |
| Kneller RW et al,1991^[10]^ | Cohort | Man | United States | 75/17633 | Dietary questionnaire/  US Department of Agriculture food composition tables | Quartile (≥4 glasses/d vs. <1 glassed /d) | dairy  Milk | 1.20(0.61-2.44)  2.40(1.10-5.04) | Year of birth and current cigarette smoking |
| Boeing H et al, 1991^[11]^ | HCC | Total | Germany | 143/579 | FFQ/German Federal Agency of Nutrition | Tertile  (III vs. I) | Dairy products  Milk  Cheese | 0.63(0.39-1.03)  1.31(0.82-2.10)  0.44(0.26-0.77) | Age, sex and hospital |
| Boeing H et al, 1991^[12]^ | HCC | Total | Poland | 741/741 | Dietary questionnaire/- | Tertile (high vs. low consumption) | Cheese | 0.92(0.67-1.26) | Age, sex, occupation, education, and residency |
| Hansson LE et, al, 1993^[13]^ | PCC | Total | Sweden | 338/680 | FFQ/- | Quartile (IV vs. I) | Whole milk  Skimmed milk  Soured milk  Cheese | 1.73(1.02-2.94)  0.77(0.53-1.12)  0.90(0.58-1.42)  0.79(0.48-1.32) | Age, gender and SES |
| Cornee J et, al,1995^[14]^ | HCC | Total | France | 92/128 | Dietary history questionnaire/  French and English food tables | Tertile (III vs. I) | Total dairy products  Milk  Hard cheese  Soft cheese  yoghurt | 1.80(0.89-3.66)  1.57(0.75-3.29)  1.48(0.74-2.96)  0.92(0.47-1.80)  0.75(0.37-1.54) | Age, sex, occupation and total energy intake |
| Harrison LE et, al 1997^[15]^ | HCC | Total | United States | 91/132 | FFQ/USDA’s food composition data tape | Quartile (IV vs. I) | Dairy | Intestinal:1.1(0.7-1.6)  Diffuse:1.2(0.7-1.9) | Caloric intake, age, gender, race, education, smoking, alcohol intake and BMI |
| Galanis DJ et, al 1998^[16]^ | Cohort | Total | Japanese residents of Hawaii, US | 108/10799 | FFQ/- | 1 or more vs. None cups/day | Milk | 1.00(0.70-1.50) | Age, years of education, Japanese place of birth, gender, smoking and alcohol intake |
| Ward MH et al, 1999^[17]^ | PCC | Total | Mexico | 220/752 | FFQ/- | Quartile (≥17 times/week vs. <5) | Dairy products  Milk  Cheese | 2.70(1.40-5.00)  2.20(1.10-4.50)  3.80(1.70-8.20) | Age, gender, total calories, chili pepper, salt, history of peptic ulcer, smoking and SES |
| Munoz N et al,2001^[18]^ | PCC | Total | Venezuela | 302/485 | FFQ/Colombian food tables | Quartile (IV vs. I) | Dairy products | 2.43(1.46-4.04) | Age, sex, tobacco, alcohol, total calories and SES |
| Kim HJ et al, 2002^[19]^ | HCC | Total | Korea | 136/136 | FFQ/Korea national food composition data | Tertile (high vs. low) | Milk and milk products | 0.68(0.34-1.36) | Sex, age, SES, family history and refrigerator use |
| Chen H et al, 2002^[20]^ | PCC | Total | United States | 124/449 | Health habits and history questionnaire/US Dietsys database | Quartile (Q4 vs. Q1) | Dairy products  Milk | 0.76(0.34-1.70)  0.86(0.39-1.90) | Age, sex, energy intake, respondent type, BMI, alcohol and tobacco use |
| Ito LS et, al 2002^[21]^ | HCC | Woman | Japan | 508/36490 | FFQ/- | Quartile (highest vs. lowest) | Milk | 0.85(0.62-1.18) | Age, year, smoking habit and family history of gastric cancer |
| Ngoan L et,al 2002^[22]^ | Cohort | Total | Japan | 116/13250 | FFQ/ | Tertile (high vs. low) | Milk products  Milk | 1.10(0.40-3.40)  0.60(0.30-1.30) | Sex, age, smoking, and other dietary factors |
| De Stefani E et, al 2004^[23]^ | HCC | Total | Uruguay | 240/960 | FFQ/ | Tertile (III vs. I) | Dairy foods | 0.89(0.59-1.33) | Age, sex, smoking, total energy intake, drinking, residence, BMI |
| Lissowska J et, al 2004^[24]^ | PCC | Total | Poland | 464/433 | FFQ/US and Polish food tables | Quartile (high: >32.9 vs. low: <18.9 times/week) | Dairy products | 0.94(0.57-1.54) | Age, sex, education, smoking, and calories |
| Khan MM et, al 2004^[25]^ | Cohort | Total | Japan | 51/3158 | Dietary factors survey/Japan dietary database | Comparison group vs. Reference group | Milk  Cheese  Yogurt | M: 1.1(0.5-2.2)  F: 1.2(0.5-3.0)  M: 1.0(0.4-3.0)  F: 1.2(0.3-5.4)  M: 1.6(0.8-3.6)  F: 0.3(0.0-2.3) | Age and smoking |
| Tokui N et, al 2005^[26]^ | Cohort | Total | Japan | 859/110792 | FFQ/Japanese food tables | 1+/day vs. None | Milk  Cheese  Yogurt | M:1.06(0.84-1.35)  F: 0.83(0.6-1.13)  M:0.79(0.39-1.61)  F: 1.18(0.52-2.69)  M:0.82(0.50-1.37)  F:0.88(0.47-1.64) | Age and sex |
| Fei SJ et, al 2006^[27]^ | HCC | Total | China | 189/567 | Dietary history questionnaire/- | Cases vs. controls | milk products | 0.69(0.52-0.91) | Not mention |
| van der Pols JC et, al 2007^[28]^ | Cohort | Total | England and Scotland | 32/4374 | Household inventory method/UK nutrition database | Quartile (Group4 vs. Group1) | Total dairy  milk | 0.81(0.09-7.34)  0.79(0.11-5.73) | Age, sex, fruit, energy and calcium intake |
| Navarro Silvera SA, et, al, 2008^[29]^ | PCC | Total | United States | 607/687 | FFQ/USDA’s food composition tables | Cases vs. controls | dairy | C:1.19(0.98-1.45)  N:1.10(0.93-1.32) | Sex, anatomic site, age, race, energy intake, income, education, BMI, smoking and drinking |
| Pourfarzi F et, al, 2009^[30]^ | PCC | Total | Iran | 217/394 | Structured questionnaire/- | ≥once/day vs. ≤2 times/week | Dairy products  cheese | 2.28(1.23-4.22)  1.16(0.54-2.51) | Gender, age, education, meat, fish, tea, garlic, family history of gastric cancer, salt intake and H. pylori. |
| Lazarevic K et, al 2010^[31]^ | HCC | Total | Serbia | 102/204 | FFQ/- | Tertile (III vs. I) | Dairy  Milk | 0.63(0.33-1.72)  5.08(1.59-10.16) | Age, sex, residence, education, meals regularity, smoking, history of cancer |
| Gao Y et, al 2011^[32]^ | PCC | Total | China | 915/1514 | Interviewer-administered questionnaire/- | Ever vs. never | Milk or dairy products | C:2.08(1.67-2.59)  N:1.69(1.29-2.21) | Age, gender, geographic region |
| Pakseresht M et, al 2011^[33]^ | PCC | Total | Iran | 286/304 | FFQ/UK food composition data set | Cases vs. Controls  (per 100g) | Dairy | 1.01(0.9-1.13) | Age, sex, education, smoking, income, using refrigerator, total energy intake, frying, H. pylori infection |
| Ko kp et, al 2013^[34]^ | cohort | Total | Korea | 166/9724 | FFQ/- | Quartile(≥1 time/day vs. never) | Dairy products | 1.30(0.83-2.06) | Age, sex, smoking, BMI, alcohol drinking, area of residence |

BMI, body mass index; SES, social economic status; M: Male; F: Female; FFQ: Food Frequency Questionnaire; HCC: Hospital-based Case-Control study; PCC: Population-based Case-control Study; C: gastric cardia adenocarcinoma; N: gastric non-cardia adenocarcinoma
